# Nonvolatile liquid anthracenes for facile full-colour luminescence tuning at single blue-light excitation

**DOI:** 10.1038/ncomms2969

**Published:** 2013-06-05

**Authors:** Sukumaran Santhosh Babu, Martin J. Hollamby, Junko Aimi, Hiroaki Ozawa, Akinori Saeki, Shu Seki, Kenji Kobayashi, Keita Hagiwara, Michito Yoshizawa, Helmuth Möhwald, Takashi Nakanishi

**Affiliations:** 1 National Institute for Materials Science (NIMS), 1-2-1 Sengen, Tsukuba 305-0047, Japan; 2 Department of Applied Chemistry, Graduate School of Engineering, Osaka University, Osaka 565-0871, Japan; 3 Department of Chemistry, Faculty of Science, Shizuoka University, 836 Ohya, Suruga-ku, Shizuoka 422-8529, Japan; 4 Chemical Resources Laboratory, Tokyo Institute of Technology, Yokohama 226-8503, Japan; 5 Department of Interfaces, Max Planck Institute of Colloids and Interfaces, Research Campus Golm, Potsdam 14476, Germany

## Abstract

Nonvolatile room-temperature luminescent molecular liquids are a new generation of organic soft materials. They possess high stability, versatile optical properties, solvent-free fluid behaviour and can effectively accommodate dopant dye molecules. Here we introduce an approach to optimize anthracene-based liquid materials, focussing on enhanced stability, fluorescence quantum yield, colour tunability and processability, with a view to flexible electronic applications. Enveloping the anthracene core in low-viscosity branched aliphatic chains results in stable, nonvolatile, emissive liquid materials. Up to 96% efficient energy-transfer-assisted tunable emission is achieved by doping a minute amount of acceptor dye in the solvent-free state. Furthermore, we use a thermoresponsive dopant to impart thermally controllable luminescence colours. The introduced strategy leading to diverse luminescence colours at a single blue-light excitation can be an innovative replacement for currently used luminescent materials, providing useful continuous emissive layers in developing foldable devices.

## Abstract (web summary)

Nonvolatile luminescent liquids are solvent-free fluids with a range of flexible electronic applications. Here, the authors show that anthracenes enveloped in branched aliphatic compounds are stable emissive liquids, with emissive and thermoresponsive properties that may be tuned by addition of dopants.

## Graphical abstract

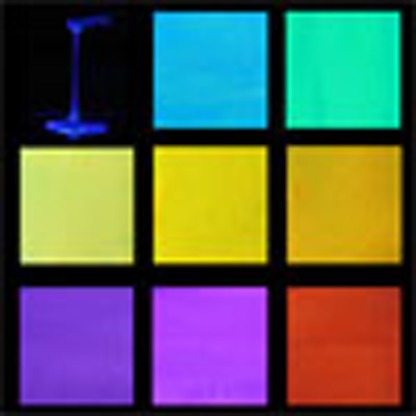

Materials with tunable colours continue to fascinate mankind, and stable, colourful and emissive materials^1^,^2^ have many potential applications. At the same time, these materials are key to new research aimed at generating low cost and energy-saving light sources^3^. Advanced organic synthetic methodologies have been extensively used to develop new chemicals with improved quantum efficiency. However, finding simple and controllable methods to process these emissive organic materials remains a major challenge^4^. In addition, π-conjugated molecules inherently aggregate, leading to optical and electronic properties that vary drastically according to the local environment of the π-conjugated core and applied conditions. Because the materialization of uninterrupted emissive layers using paintable organic liquids has yet to be realized, reliable emissive materials that can maintain continuous active layers when bent and folded^5^ are required for the future prospects of flexible devices^6^. Newly developed, stable and emissive liquid materials could be potential candidates for this goal. It is also important to simply demonstrate how a full range of coloured emission can be obtained without much synthetic effort, for example, by doping (dissolving) minute amounts of dyes into the emissive liquid.

Small aromatic molecules such as anthracenes are extensively studied for organic electronics because of the charge-carrier mobility of their ordered assemblies^7^,^8^, high quantum yield and blue emissive features^9^. Despite this, their sensitivity to air and light^10^, and the inherent difficulty of preparing homogeneous thin films from their crystalline assemblies, while retaining high quantum yields^11^ and a diversity in luminescence colour^12^, are still major drawbacks. In this context, a new approach that can overcome all these issues is highly desirable. A nonvolatile, low-viscosity and highly emissive room-temperature liquid material is a promising candidate. Recently, we prepared oligo(*p*-phenylenevinylene)-based blue-emitting solvent-free liquids by isolating the π-conjugated oligo(*p*-phenylenevinylene) core using bulky and soft aliphatic chains. These were used as a matrix/solvent to produce white light-emitting paints^13^. The advantage of functional molecular liquids is their ability to retain the features of isolated optoelectronically active π-conjugated moieties in the bulk fluid state. In this respect, there is a clear difference between the above-mentioned liquids^13^ and other amorphous organic fluids that have been obtained by merely lowering the glass-transition temperature^14^,^15^,^16^.

Here, by attaching branched low-viscosity aliphatic chains to the anthracene core, we report nonvolatile, blue-emitting and highly stable room-temperature liquid anthracenes into which acceptor dyes can be doped to tune the luminescence colour. The dye-doped anthracene liquid exhibits a variance in luminescence covering almost the entire visible range. This ease of luminescence colour tuning and the liquid features of the blends pave a practical way towards reliable emissive materials capable of forming facile coatings and acting as a continuous active layer in flexible devices.

## Results

### Synthesis and characterization of anthracene liquids

**Figure 1:**
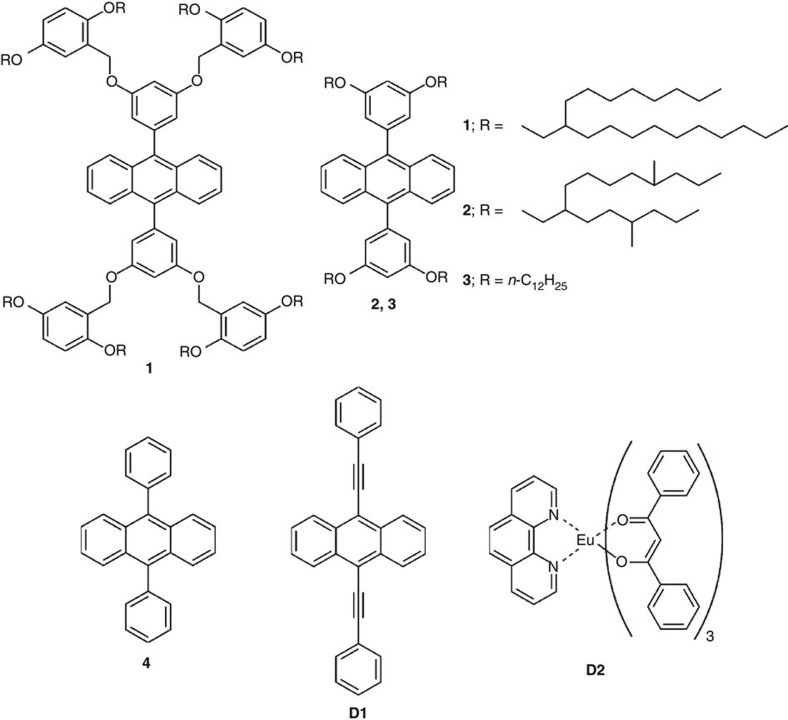
Molecular structure of anthracenes and dopants. The figure shows the chemical structure of anthracenes (1–**4**) and dopants (**D1** and **D2**) used in this study.

This study presents a versatile approach that makes use of the high photostability, fluorescence quantum yield and colour tunability of anthracene derivatives to design novel light-emitting liquid materials. As depicted in Fig. 1, we have synthesized two new anthracene derivatives (1 and 2). The anthracene derivatives 1 and 2 were obtained as yellowish transparent viscous fluids at room temperature, whereas the reference molecule 3 was a pasty solid. All anthracenes were unambiguously characterized by ultraviolet-visible absorption, steady-state fluorescence, ^1^H, ^13^C NMR and matrix-assisted laser desorption ionization time-of-flight mass spectrometry (MALDI–TOF MS).

**Figure 2:**
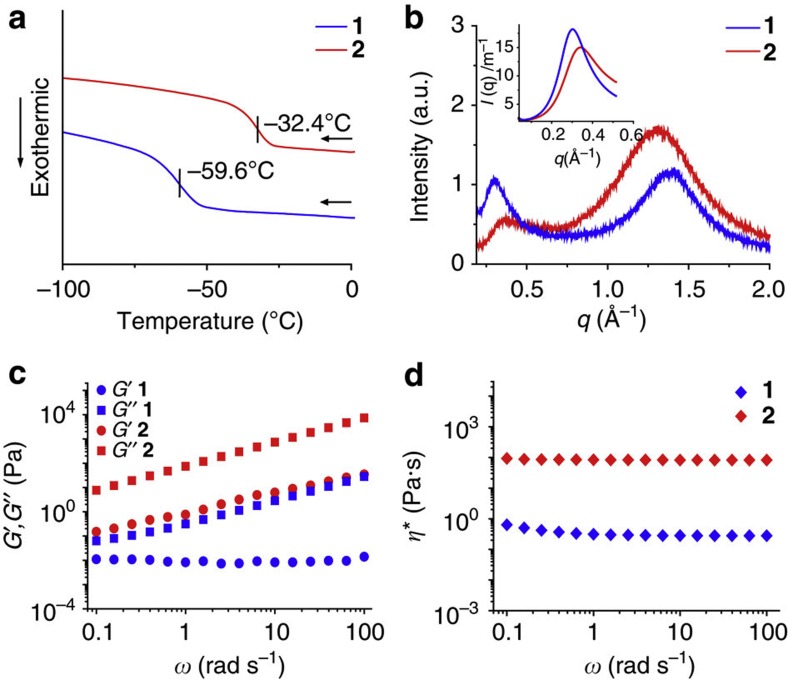
Characterization of solvent-free room-temperature liquid anthracenes. (**a**) DSC thermograms in the cooling trace showing the glass-transition temperatures (*T*_g_). (**b**) XRD diagrams with small-angle X-ray scattering profiles as insets, variation of (**c**) storage modulus (*G′*)(circles) and loss modulus (*G″*) (squares), as well as (**d**) complex viscosity (η*), (diamonds) versus angular frequency on double logarithmic scale, of 1 (blue markers) and 2 (red markers).

The ^1^H NMR (Supplementary Figs S1 and S2) and thermogravimetric analysis (TGA) (Supplementary Fig. S3) indicated an absence of residual solvent in the bulk fluid sample. Swallow-tail type aliphatic chains attached to a dendritic skeleton of the phenyl substituent and hyperbranched aliphatic chains without the dendritic skeleton were used in [Chem-struct co1] and 2 respectively, to ensure significant fluidity^17^ at room temperature. On the other hand, linear aliphatic chains present in 3 imparted solid features at room temperature, emphasising the importance of branched alkyl chains in obtaining solvent-free liquids at room temperature. In the cooling trace of the differential scanning calorimetry (DSC) thermogram, 1 exhibits a glass-transition temperature (*T*_g_) of −59.6 °C (molar heat capacity *C*_mol_; 1955.1 J mol^−1^ K^−1^), which is increased to −32.4 °C (*C*_mol_; 389.6 J mol^−1^ K^−1^) for 2 (Fig. 2a). Both molecules exist in the solvent-free fluid state over a wide range of temperatures up to around 350 °C (Supplementary Fig. S3). In the X-ray diffraction (XRD) analysis, 1 and 2 both exhibit a halo in the wide angle region corresponding to an average distance between the molten aliphatic chains of 4.5 Å for 1 (*q*=1.4 Å^−1^) and 4.8 Å for 2 (*q*=1.3 Å^−1^), respectively (Fig. 2b, Supplementary Fig. S4). The disordered anthracene core–core distance of around 21 Å for 1 and 17 Å for 2 appeared as a broad halo at smaller *q* in both the XRD (*q*=0.30 Å^−1^ for 1 and 0.36 Å^−1^ for 2) and small-angle X-ray scattering (inset of Fig. 2b) (*q*=0.30 Å^−1^ for 1 and 0.34 Å^−1^ for 2) results. This distance is larger for 1 than for 2 because of the presence of bulkier substituents around the anthracene core (for example, schematic illustration in Supplementary Fig. S4c). In rheology experiments, the loss modulus *G″* is found to be higher than the storage modulus *G*′ over the measured angular frequency range of *ω*=0.1–100 rad s^−1^ and for a shear strain of *γ*=0.1% (Fig. 2c), indicating liquid behaviour. In addition, a significant reduction in complex viscosity (η*) was observed from 84.0 (2) to 0.28 Pa·s (1) at *ω*=10 rad s^−1^ (Fig. 2d, Supplementary Table S1). In conclusion, ^1^H NMR, TGA, DSC, XRD, small-angle X-ray scattering and rheology experiments confirmed the solvent-free liquid characteristics of anthracenes 1 and 2.

### Photostability studies

**Figure 3:**
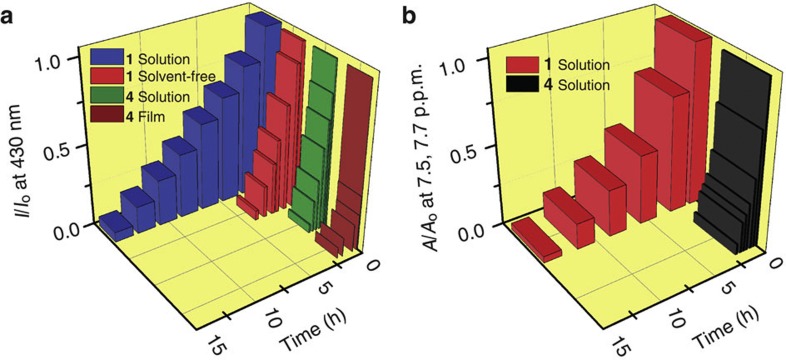
Photostability studies. Comparison of the variation of (**a**) fluorescence intensity at 430 nm in solvent-free states of 1 and **4** (Solvent-free, Film) supported on a quartz plate and in dichloromethane solution (Solution) (*c*=1 × 10^−4^ M, *l*=1 mm, *λ*_ex_=375 nm) of 1 and **4**. (**b**) Variation of the ^1^H NMR peak area of aromatic protons of 1 (7.7 p.p.m.) and **4** (7.5 p.p.m.) in dichloromethane-*d*_2_.

Steric hindrance via soft and bulky tail attachment can disturb intermolecular π–π interactions among adjacent anthracene units^18^,^19^ in the solvent-free liquid state and improve the photostability. To demonstrate this, photo-stability studies have been carried out by irradiating the samples at 365 nm (100 W m^−2^) using a Xe lamp. In comparison with cast thin film of 9,10-diphenylanthracene **4** (Fig. 1, Supplementary Figs S5 and S6), the liquid anthracenes exhibited a remarkable improvement in stability (Supplementary Fig. S7). As shown in Fig. 3, 1 was optically active for up to 4 h under irradiation in the solvent-free state and 15 h in dilute dichloromethane solution. In comparison, the characteristic absorption (375 nm, Supplementary Fig. S5a) and emission (430 nm, Fig. 3a) peaks of the anthracene chromophore of **4** in solution had completely disappeared after just 2 h (Supplementary Figs S5 and S6). This could be attributed to conversion of the anthracene unit into 9,10-endoperoxides upon irradiation^20^. The ^1^H resonance characteristics of the parent anthracene protons exhibited significant changes upon irradiation (Supplementary Fig. S8). The formation of a photo-oxidized 9,10-endoperoxide on 1 was confirmed by the appearance of a peak at 84.5 p.p.m. in ^13^C NMR analysis (Supplementary Fig. S9a)^20^,^21^. The 9,10-endoperoxide was also formed by **4** under the same experimental conditions (Supplementary Fig. S9b). The failure of the [4+2] Diels–Alder cycloaddition^22^ between fullerene C_60_ and liquid anthracenes also supports the enhanced stability of these materials. The pronounced stability of liquid anthracenes is directly related to the enveloping of the anthracene core by bulky branched alkyl chains to reduce the attack by oxygen and dimerization of the core.

### Optical features of monomer-like bulk liquids

**Figure 4:**
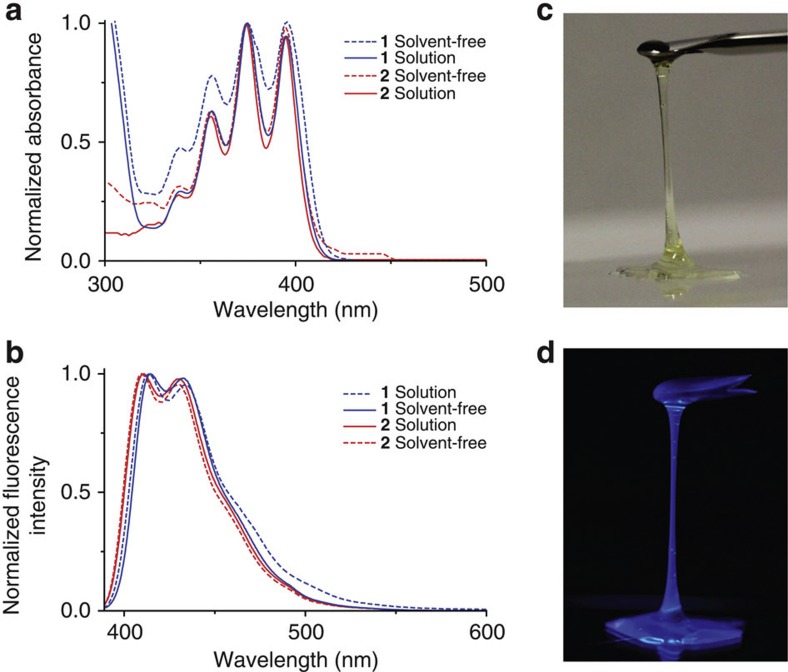
Optical features of liquid anthracenes. Comparison of the normalized (**a**) absorption and (**b**) fluorescence spectra of 1 and 2 in a solvent-free bulk liquid state (solvent-free) supported on a quartz plate at room temperature and in dichloromethane solution (Solution) (*c*=5 × 10^−5^ M, *l*=1 mm, *λ*_ex_=375 nm). Photographs of 2 under (**c**) visible and (**d**) ultraviolet light (365 nm).

To understand how effectively the core is isolated in the solvent-free liquids, absorption and emission spectra of 1 and 2 (Fig. 4, Supplementary Fig. S10) were compared in the solvent-free state and dilute solution. Intermolecular interactions of the anthracene core are almost completely prevented, and monomeric features identical to those in dilute dichloromethane solutions are retained in the dense bulk liquid state. Despite a longer wavelength tail appearing in the absorption spectra in the solvent-free state, which is likely to be caused by scattering from the surface, the emission features are identical in both cases. The yellowish, transparent liquid of 2 under visible light (Fig. 4c) exhibits a strong blue emission under ultraviolet light (*λ*_ex_=365 nm, Fig. 4d). The blue-emitting anthracene liquids (1 and 2) exhibit high absolute fluorescence quantum yields of ca. 55% in solvent-free thin films under ambient conditions (Supplementary Table S1). These are somewhat lower than the values obtained for the dilute dichloromethane solutions (Supplementary Table S2), but still relatively high for solid or film states of anthracenes^23^. Values for the absolute fluorescence quantum yield at liquid nitrogen temperature and refractive index values of the derivatives are summarized in Supplementary Table S1. The ultraviolet-visible absorption, steady-state fluorescence and photoconductivity measurements (Supplementary Fig. S11) highlight the importance of our design strategy to yield room-temperature liquids by enveloping each core with branched alkyl chains to reduce the intermolecular core–core interactions. This core isolation strategy enables the intrinsic monomeric characteristics of a π-conjugated core unit to be achieved in bulk, solvent-free liquid matter.

### Energy transfer with minute doping

In contrast to previously reported organic luminescent materials that have substantially reduced quantum efficiency in the solvent-free solid or film state, high quantum yields have been achieved by these luminescent solvent-free liquids. A long-standing problem with organic materials has thereby been solved through this alternative route. A further development is to manipulate the luminescence colour in a facile and cost-effective way. Recently, organic materials with tunable luminescence behaviour have gained much attention^24^,^25^,^26^,^27^,^28^,^29^,^30^. Among those systems, energy transfer assisted light harvesting assemblies with tunable emission properties have been widely studied^28^,^29^,^30^. However, in most other cases the ability to effectively tune the colour with only a very small mol% of acceptor dopants in solvent-free conditions is rather limited. To control the emission features of the anthracene liquids, minute amounts of different solid dopants were used, namely 9,10-bis(phenylethynyl)anthracene (dopant-1, **D1**) and tris(1,3-diphenyl-1,3-propanedionato)(1,10-phenanthroline)europium(III) (dopant-2, **D2**) (Fig. 1). Owing to its low viscosity in the solvent-free state, 1 was used as the liquid matrix into which **D1** and **D2** could be dissolved by simply blending with a spatula. The spectral overlap integral between the emission of donor 1 and the absorption of acceptor **D1** was found to be *J*(*λ*)=8.86 × 10^14^ M^−1^ cm^−1^ nm^4^, indicating that **D1** can be a suitable acceptor for energy transfer from 1 (Supplementary Fig. S12)^31^,^32^.

**Figure 5:**
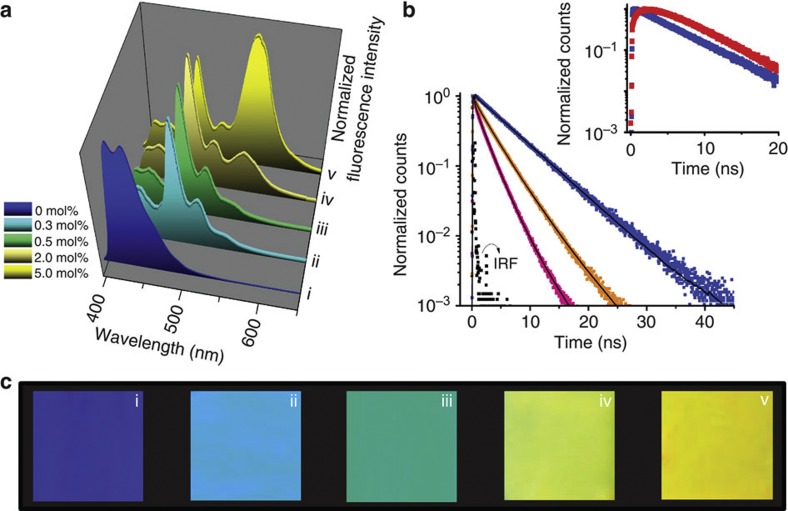
Energy-transfer-assisted tunable emission. (**a**) Normalized emission spectral changes of 1 upon increasing the concentration of **D1** (*λ*_ex_=350 nm). (**b**) Emission lifetime decay profile of 1 (blue squares) (*λ*_ex_=377 nm) in the presence of 0.3 (orange squares) and 0.5 mol% (pink squares) of **D1** monitored at 430 nm supported on quartz plate, and inset shows the decay profiles of **D1** (0.3 mol%) in the matrix of 1 (red squares) and **D1** alone (blue squares), monitored at 475 nm (*λ*_ex_=377 nm). IRF corresponds to Instrument Response Function. (**c**) Photographs of the luminescent thin films of 1 (i) and 1 doped with **D1** (ii–v), which are correlated to the emission spectra of (**a**).

In the energy transfer system, 95% fluorescence quenching of 1 and efficient energy transfer to **D1** was observed for only 0.3 mol% of **D1** (Fig. 5a, Supplementary Fig. S12) when excited at 350 nm, where the **D1** absorption is at a minimum. The efficiency of energy transfer is increased with increasing mol% of **D1**, for example, at 1.0 mol% the energy transfer efficiency was found to be 96% (Supplementary Fig. S13). The energy transfer rate was calculated to be 3.75 × 10^9^ s^−1^ with a Förster distance (*R*_0_), at which the energy-transfer efficiency becomes 50%, of 44 Å. Under these conditions, 100% fluorescence enhancement was observed for **D1** at 475 nm, when compared with the direct excitation of **D1** at 440 nm in the donor matrix (Supplementary Fig. S13). The excitation spectrum of the energy-transfer system monitored at 475 nm revealed a relatively low contribution from the direct excitation of **D1** (Supplementary Fig. S13). Interestingly, at higher mol%, acceptor **D1** tends to aggregate in the donor scaffold, and energy is transferred to both monomers and aggregates of **D1**.

The energy transfer was further confirmed by fluorescence lifetime decay profiles of 1 monitored at 430 nm in the absence and presence of **D1** (Fig. 5b). The fluorescence decay profile of 1 exhibited a biexponential decay with components having lifetimes of 5.8 (98%) and 0.35 ns (2%). As the concentration of **D1** is increased, a triexponential decay with lifetimes of 4.3 (33%), 0.13 (64%) and 0.22 ns (3%) at 0.3 mol% of **D1**, and further 3.5 (18%), 1.84 (75%) and 0.29 ns (7%) at 0.5 mol% of **D1**, were observed. Such a decrease of the anthracene donor lifetime in presence of the acceptor is direct evidence of the fluorescence resonance energy transfer^28^. As additional evidence for energy transfer, a temporal growth in the emission of the acceptor when excited at 377 nm into the donor matrix (Fig. 5b, inset) was observed. The requirement of just 0.3 mol% of acceptor for efficient energy transfer is the lowest quantity reported for any supramolecular energy transfer system^30^. One possible reason for the increased efficiency at low acceptor loading could be the presence of small clusters in the monomer-like bulk liquid donor. Such an energy transfer in disordered aggregates is well documented^33^,^34^. The structural similarity and ease of trapping of the acceptor molecules in these small clusters results in efficient energy transfer. Another interesting aspect related to energy transfer is the fluorescence colour variation as visualized in Fig. 5c. Upon increasing the concentration of **D1**, the donor fluorescence is sequentially quenched and the blue emission colour from 1 (Fig. 5c (i)) changed to cyan at 0.3 mol% of **D1** (Fig. 5c (ii)). The colour was completely changed to green at 0.5 mol% (Fig. 5c (iii)), to greenish yellow at 2 mol% (Fig. 5c (iv)) and finally to yellow at 5 mol% (Fig. 5c (v)). At high doping mol%, a fluorescence combination of donor, monomer and aggregate of the acceptor was observed (Fig. 5a). Such a wide-range luminescence colour tuning using a minute amount of dopant has not been reported so far, and it could be attributed to the enhanced aggregation tendency of **D1** in the donor matrix.

### Thermoresponsive features of solvent-free composites

**Figure 6:**
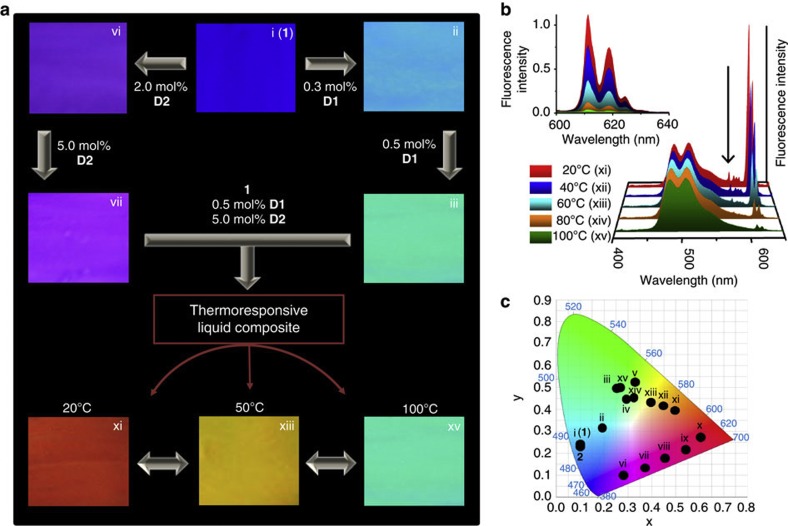
Tunable emission colours from liquid composites. (**a**) Photographs of the luminescence colour tunability and thermoresponsive feature of the composites of 1, **D1** (0.5 mol%) and **D2** (5 mol%). (**b**) Thermoreversible luminescence spectral changes of the composite of 1, **D1** and **D2**, supported on a quartz plate (*λ*_ex_=375 nm) upon heating; inset shows the corresponding emission changes from 600 to 640 nm. (**c**) Commission internationale de l′éclairage coordinate values of the overall colour tunability achieved by doping of 1 with **D1** and/or **D2**, as well as with temperature. The Commission internationale de l′éclairage coordinate also includes 2. The indications of ii–v are from the composites of 1 and **D1** (Fig. 5), vi–x are from the composites of 1 and **D2** (Supplementary Fig. S14a) and xi–xv are from composites of 1, **D1** and **D2** (**b**).

To extend the tunable emission features, we monitored the luminescence changes of 1 by adding **D2** (Supplementary Fig. S14, composites denoted as vi–x). Upon addition of **D2**, the intensity of the 0-0 vibronic band in the fluorescence spectrum of 1 was decreased with an accompanying increase in the **D2** emission intensity when excited at 375 nm, indicating the possibility of a weak energy transfer from 1 to **D2**. A violet colour at 2 mol% (Fig. 6a (vi)) and purple colour at 5 mol% (Fig. 6a (vii)) of **D2** was observed. A combination of 0.5 mol% **D1** and 5 mol% **D2** resulted in a red emission (Fig. 6a (xi)). The temperature dependent emission of europium complexes (for example, **D2**) has been extensively studied and this property has been used for various sensing applications^35^. Here the thermoresponsivity of **D2** enables luminescence modulation of the liquid composite. On increasing the temperature, the red emission colour was changed to yellow at around 50 °C (Fig. 6a (xiii)) and to emerald green after a further increase in temperature (Fig. 6a (xv)), which is mostly contributed by the luminescence of **D2**. The fluorescence spectra upon heating (Fig. 6b) and cooling (Supplementary Fig. S14) between 20 and 100 °C, exhibited reversible changes, especially between 600 and 640 nm.

The thermoresponsive liquid composite behaves as a thermometer with reversible luminescence colour variation^36^. Because the emission centred at 610 nm is thermally gated, the liquid composite exhibits temperature controlled luminescence tuning with attractive colours. Although the triexponential lifetime of 1 in the composite showed a drastic decrease to 2.1 ns (39%), 0.9 ns (50%) and 0.24 ps (11%) (Supplementary Fig. S14), a clear picture of the electronic communication between 1, **D1** and **D2** is not available because there is a possibility of direct excitation of **D2** at 375 nm. However, the excitation spectrum of the composite monitored at 610 nm reveals contributions from 1 and **D1** (Supplementary Fig. S14)^37^. Hence, the thermoresponsive features of **D2** enabled manipulation of various luminescence colours (Fig. 6c, Supplementary Table S3). The resulting tunable composite system obtained by mixing two dopants with liquid 1 in specific ratios retained fluid characteristics (Supplementary Fig. S15), making room for further diversification of colour obtainable at single ultraviolet wavelength excitation.

## Discussion

Blue-emitting, nonvolatile, low-viscosity, room-temperature anthracene liquids, devoid of various disadvantages of the currently available anthracene-based organic functional materials, have been synthesized. The liquid anthracenes have a high fluorescence quantum yield and exhibit improved photostability under ambient conditions. The very low viscosity of these emissive liquids allows doping of other emissive and thermoresponsive components into the liquid matrix, leading to a tunable luminescence colour. The most efficient energy transfer from an anthracene unit to any dopant and the controllable thermoresponsive luminescence colour changes in the absence of solvent are both new phenomena. The liquid composite with a range of reversible luminescence colours covering almost the whole visible spectrum can also be used as a colour-indicating thermometer. This facile luminescence variation has strong advantages over other reported methods using either complex synthesis or multicomponent systems^29^. Our rational design allows the production of enriched liquid matter, which provides technological potential for a continuous active layer in flexible materials. We therefore believe that these materials will be promising candidate with many opportunities to design low-cost, environmentally-friendly, flexible, foldable luminescent products.

## Methods

### Materials

Unless otherwise stated, all starting materials and reagents were purchased from commercial suppliers and used without further purification. The hyperbranched alcohol Fine Oxocol 180N, containing isomers, was gifted by Nissan Chemical Industries, Ltd., Japan, and converted to the bromo derivative. Tris(1,3-diphenyl-1,3-propanedionato)(1,10-phenanthroline)europium(III) and 9,10-bis(phenylethynyl)anthracene (Aldrich) were used as dopants.

### Synthesis and characterization of 1–3

In a general synthetic procedure (Supplementary Methods), a mixture of 9,10-bis(3,5-dihydroxyphenyl)anthracene (2.5 mmol), corresponding alkyl and alkoxybromides (12.6 mmol), and potassium carbonate (20.28 mmol) and potassium iodide (0.5 mmol) in anhydrous dimethylformamide (30 ml) was refluxed at 115 °C for 20 h. The progress of the reactions was monitored by thin-layer chromatography (TLC), and after the completion of the reaction, the reaction mixture was cooled to room temperature, excess chloroform was added and washed with distilled water. The organic layer was washed with brine, dried over anhydrous sodium sulphate and the solvent was evaporated under reduced pressure. The purification was carried out by column chromatography (*n*-hexane/chloroform) over silica gel yielding pure anthracenes 1 and 2 as yellow liquids and 3 as a white pasty solid. The final purification has been carried out by recycling HPLC using chloroform as the solvent. 1: Transparent yellowish liquid (yield, 50%). *T*_g_: −59.6 °C; TLC (CHCl_3_: *n*-hexane, 85:15 (v/v)): *R*_f_=0.49; ^1^H NMR (400 MHz, CDCl_3_): *δ*=7.75–7.80 (*m*, 4H, Ar), 7.30–7.36 (*m*, 4H, Ar), 7.07–7.12 (*s*, 4H, Ar), 6.85–6.88 (*s*, 2H, Ar), 6.80–6.84 (*m*, 8H, Ar), 6.74–6.77 (*s*, 4H, Ar), 5.11–5.16 (*m*, 8H, O-C*H*_2_-Ar), 3.75–3.85 (*m*, 16H, OCH_2_), 1.70–1.85 (*m*, 8H, C*H*(CH_2_)_3_), 1.05–1.53 (*m*, 256H, CH_2_), 0.80–0.95 (*m*, 48H, CH_3_); ^13^C NMR (75 MHz, CD_2_Cl_2_): *δ*=154.0, 151.3, 141.6, 137.6, 130.2, 127.6, 127.1, 125.6, 115.9, 114.7, 112.9, 110.9, 101.9, 71.9, 65.7, 38.6, 32.4, 31.8, 30.2, 27.3, 23.2, 14.4; MALDI–TOF MS (matrix-dithranol): calculated for C_214_H_362_O_12_ 3,127.16, found 3,125.81 [M^+^]. 2: Transparent yellowish liquid (yield, 65%). *T*_g_: −32.4 °C; TLC (CHCl_3_: *n*-hexane, 90:10 (v/v)): *R*_f_=0.53; ^1^H NMR (400 MHz, CDCl_3_): *δ*=7.60–7.85 (*m*, 4H, Ar), 7.36–7.42 (*m*, 4H, Ar), 6.66–6.67 (*s*, 2H, Ar), 6.60–6.65 (*s*, 4H, Ar), 3.84–4.05 (*m*, 8H, OCH_2_), 1.78–2.05 (*m*, 4H, C*H*(CH_2_)_3_), 1.25–1.45 (*m*, 80H, CH_2_), 1.05–1.22 (*m*, 8H, (CH_3_)C*H*(CH_2_)_2_, 0.78–0.89 (*m*, 48H, CH_3_); ^13^C NMR (75 MHz, CD_2_Cl_2_): *δ=*141.5, 137.9, 130.2, 127.6, 125.7, 110.4, 101.4, 71.9, 40.1, 39.9, 39.1, 38.6, 37.9, 37.8, 37.7, 37.5, 37.3, 37.2, 36.8, 35.1, 35.0, 34.9, 34.0, 33.4, 33.2, 32.5, 32.3, 32.2, 30.9, 30.8, 30.7, 30.5, 30.2, 30.0, 29.9, 29.8, 29.5, 28.1, 27.6, 26.1, 24.8, 23.7, 23.6, 23.5, 23.2, 22.9, 20.4, 20.0, 19.5, 19.4, 17.1, 16.9, 16.2, 15.0, 14.7, 14.5, 14.4, 14.3, 14.2, 12.6, 12.5, 11.7, 11.5, 11.2; MALDI–TOF MS (matrix-dithranol): calculated for C_98_H_162_O_4_ 1,404.33, found 1,403.40 [M^+^]. 3: White pasty solid (yield, 57%), mp: 14–15 °C; TLC (CHCl_3_: *n*-hexane, 95:5 (v/v)): *R*_f_=0.52; ^1^H NMR (400 MHz, CDCl_3_): *δ*=8.07–8.09 (*m*, 4H, Ar), 7.78–7.84 (*m*, 4H, vinylic), 7.33–7.38 (*s*, 2H, Ar), 6.61–6.70 (*s*, 4H, Ar), 3.97–4.03 (*m*, 8H, OCH_2_), 1.77–1.85 (*m*, 8H, CH_2_), 1.65–1.73 (*m*, 8H, CH_2_), 1.43–1.50 (*m*, 8H, CH_2_), 1.22–1.41 (*m*, 64H, CH_2_), 0.87–0.93 (*m*, 12H, CH_3_); ^13^C NMR (75 MHz, CD_2_Cl_2_): *δ=*161.8, 161.1, 156.1, 141.6, 137.8, 130.2, 127.6, 125.7, 110.4, 101.3, 68.9, 68.5, 64.6, 32.5, 30.2, 29.7, 29.3, 29.1, 26.6, 26.3, 26.3, 23.2, 14.4; MALDI–TOF MS (matrix-dithranol): calculated for C_74_H_114_O_4_ 1,067.69, found 1,067.12 [M^+^].

### Measurements

^1^H NMR (600 MHz) spectra were measured with a Bruker Biospin DRX-600 spectrometer, and ^13^C NMR (75 MHz) spectra were recorded on a JEOL model AL300 spectrometer using TMS as internal standard. MALDI–TOF MS were obtained by a Shimadzu AXIMA-CFR Plus station using dithranol as the matrix. Ultraviolet-visible absorption and fluorescence spectra in solution and solvent-free liquid states were recorded on a Hitachi U-2910 spectrophotometer and a JASCO FP-8300 spectrofluorometer, respectively. The absolute quantum yields were calculated using a Hamamatsu C9920-02G instrument. Time-resolved fluorescence lifetime measurements were carried out by using time-correlated single-photon counting lifetime spectroscopy system HORIBA FluoroCube 3000U-UltraFast-SP spectrophotometer (*λ*_ex_=377 nm). The quality of the fit has been judged by goodness-of-fit parameters such as *χ*^2^ test (<1.2), as well as the visual inspection of the residuals. DSC and TGA were performed with a TI instruments DSC Q2000 and SII TG/DTA 6200, respectively. Refractive index measurements were carried out at 20 °C by using a Kyoto Electronics RA-600 refractometer using light-emitting diode Na-D 589.3 nm. XRD analysis was carried out using a Rigaku RINT ULTIMA+powder diffractometer with a copper K-alpha source. The rheology experiments were carried out using an Anton Paar Physica MCR301 rheometer at 20 °C by using about 1 g of the liquid samples. The irradiation of the samples at 365 nm for the stability test has been carried out using an ASAHI spectra LAX-102 compact xenon light source.

### Photoconductivity measurements

The flash-photolysis time-resolved microwave conductivity measurement was carried out using a X-band (9 GHz) microwave circuit at low power (approximately 3 mW) and a nanosecond laser irradiation at 355 nm with photon density of 1.8 × 10^16^ photons cm^−2^ pulse^−1^. Flash-photolysis time-resolved microwave conductivity samples were prepared by pasting the samples on a quartz plate. The obtained transient conductivity (Δ*σ* in S m^−1^) was converted to the product of the quantum yield: *φ* and the sum of charge-carrier mobilities: Σμ(=μ_+_+μ_−_), by *φ*Σμ=Δ*σ* (*eI*_0_*F*_light_)^−1^, where *e*, *I*_0_ and *F*_light_ are the unit charge of a single electron (in C), incident photon density of excitation laser (in m^−2^) and a correction (or filling) factor (in m^−1^), respectively. *F*_light_ was calculated by taking into consideration the geometry and optical properties of the sample such as the size, laser cross-section and absorption of the excitation laser.

### Samples for optical measurements

Thin solvent-free liquid pastes were prepared using spatula on a quartz substrate for the fluorescence and optical absorption measurements.

### Preparation of liquid composites

Finely powdered dopants (**D1** and **D2**) were added to liquid anthracene (1) in the required molar ratio in a watch glass and blended using a spatula. Mixing of the components for around 1 min yielded the liquid composites as a yellow/orange pasty liquid, depending on the molar ratio of the dopant. The resulting composites were optically characterized by absorption and fluorescence spectroscopies.

### Samples for photo-stability NMR experiments

The ^1^H and ^13^C NMR measurements were carried out after irradiation of 5 and 20 mg of compounds per 1 g CD_2_Cl_2_ in the NMR tube. The irradiation time was optimized to be 14 h (1), 2 h (**4**) for ^1^H and 100 h (1, **4**) for ^13^C NMR experiments.

## Author contributions

S.S.B. and T.N. designed the work, carried out research, analysed data and wrote the paper. J.A. and H.O. helped to characterize the molecules. M.J.H. contributed to small-angle X-ray scattering and NMR analysis. A.S. and S.S. performed the time-resolved microwave conductivity studies and fluorescence lifetime experiments. K.K., K.H., M.Y. and H.M. were involved in the work discussion and T.N. is the principal investigator. All authors discussed the results and commented on the manuscript.

## Additional information

**How to cite this article:** Babu, S. S. *et al.* Nonvolatile liquid anthracenes for facile full colour luminescence tuning at single blue-light excitation. *Nat. Commun.* 4:1969 doi: 10.1038/ncomms2969 (2013).

## Supplementary Material

Supplementary Information Supplementary Figures S1-S15, Supplementary Tables S1-S3 and Supplementary Methods
