# Supplementary material for: Nonvolatile liquid anthracenes for facile full-colour luminescence tuning at single blue-light excitation
Source: Nat Commun. 2013 Jun 5;4:1969. doi: 10.1038/ncomms2969 (PMC3709479; doi:10.1038/ncomms2969)
Supplement: Supplementary Information — Supplementary Figures S1-S15, Supplementary Tables S1-S3 and Supplementary Methods [file ncomms2969-s1.pdf]

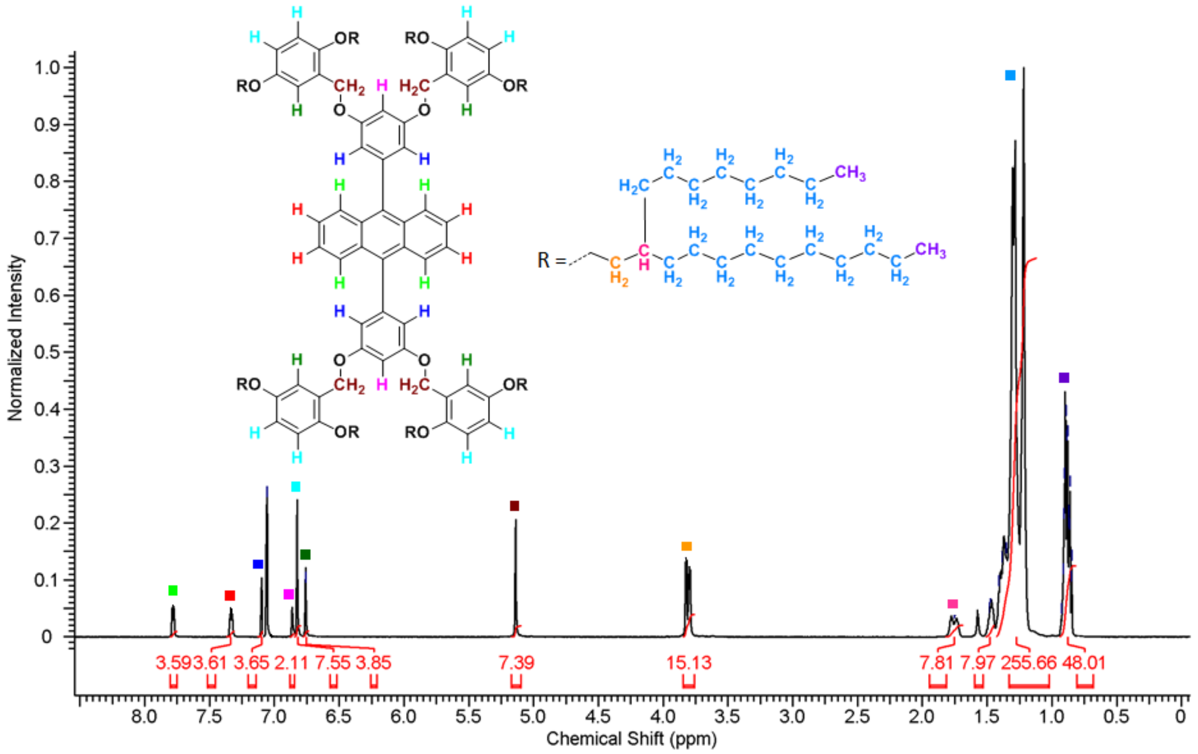

Supplementary Figure S1 | ^{1}H NMR characterization of a solvent-free fluid 1. ^{1}H NMR spectrum of **1** in $CDCl_3$, after evaporating from CH_2Cl_2 and drying under vacuum. The absence of peaks corresponding to CH_2Cl_2 in the NMR spectrum clearly states that the fluid characteristic is the inherent behaviour of the single liquid component, and not due to the trapped solvent molecules.

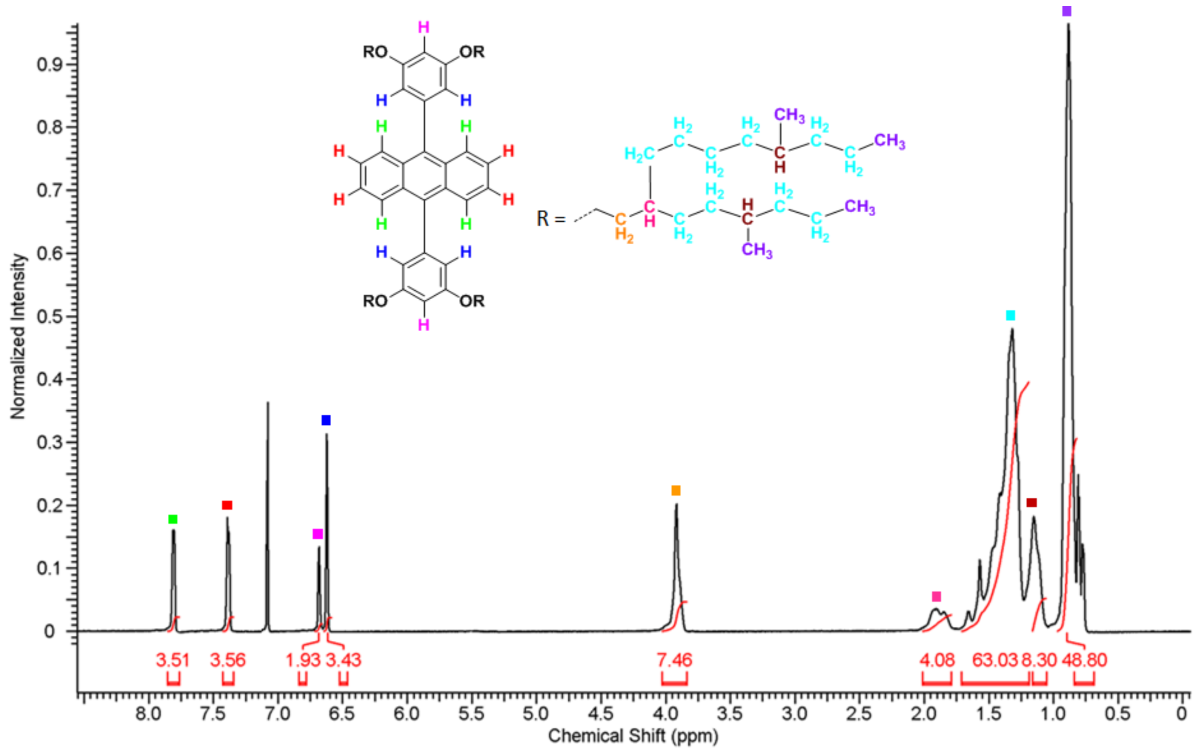

Supplementary Figure S2 | ¹H NMR characterization of a solvent-free fluid 2. ¹H NMR spectrum of **2** in $CDCl_3$, after evaporating from CH_2Cl_2 and drying under vacuum. The absence of peaks corresponding to CH_2Cl_2 in the NMR spectrum clearly states that the fluid characteristic is the inherent behaviour of the single liquid component, and not due to the trapped solvent molecules.

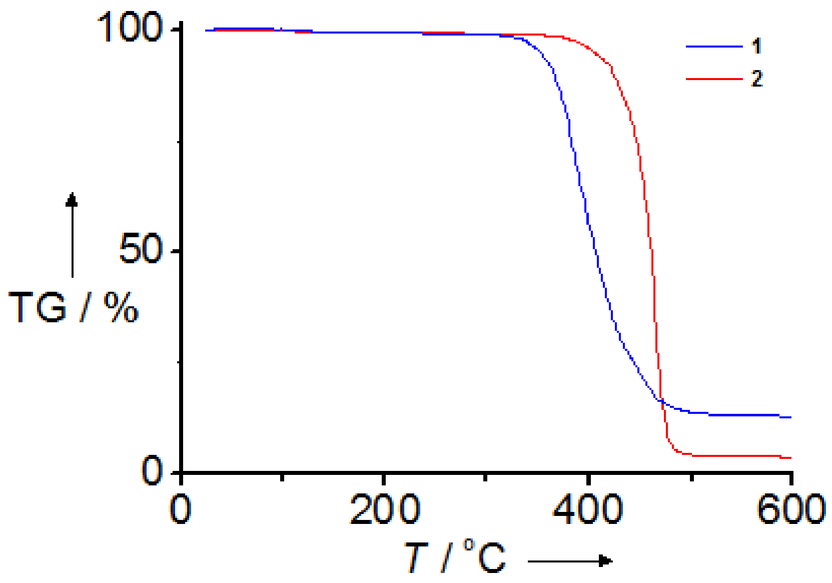

Supplementary Figure S3 | Thermogravimetric characterization of solvent-free fluids. Thermogravimetric analysis of liquid anthracenes **1** and **2**. The mass loss% at 300 ˚C was found to be 1.13% (**1**) and 0.77% (**2**).

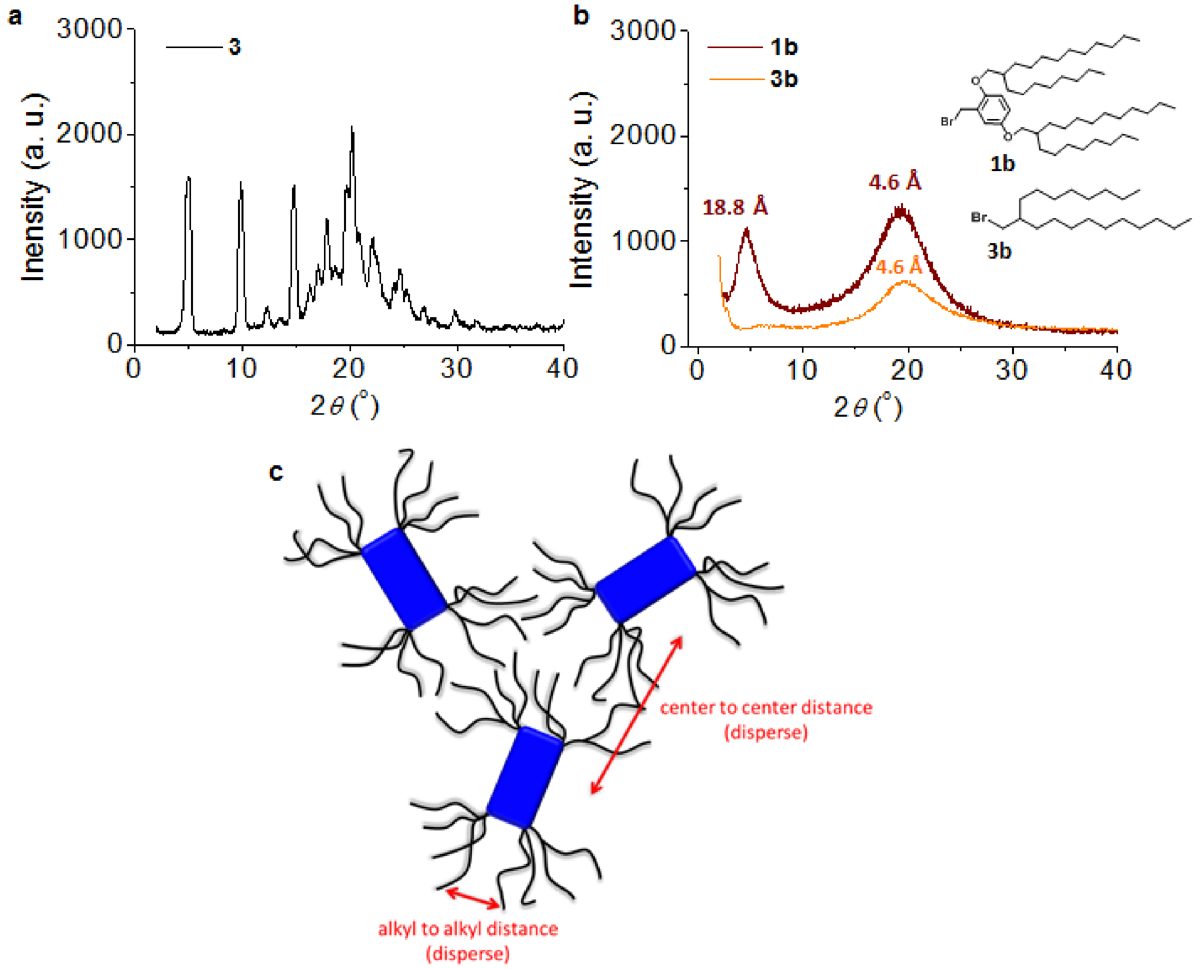

Supplementary Figure S4 | X-ray diffraction studies. XRD diagrams of (**a**) **3** and (**b**) **1b** and **3b**. (**c**) Schematic illustration of the distance information corresponding to XRD analysis of **1** and **2**. XRD of the precursor molecules indicates that in the case of **3b** only one halo corresponding to branched alkyl chains in a molten state is present, whereas when it is equipped on a benzyl bromide, **1b,** one more additional halo from the aromatic moiety appears. It clearly indicates that the two halos in the case of liquid anthracenes (**1** and **2**) correspond to the average distance of the aromatic and aliphatic moieties of the molecules in their molten phases. Compound **3**, on the other hand, exhibits crystalline reflexes due to its higher organization in the solid state.

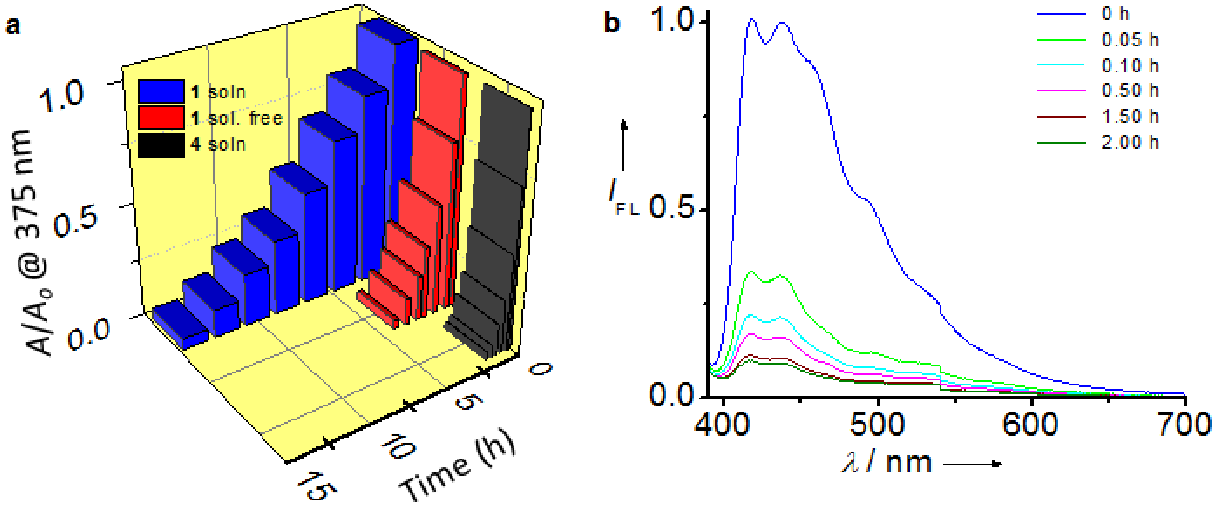

Supplementary Figure S5 │ Photostability of anthracenes. (**a**) Comparison of the variation of absorbance at 375 nm in the solvent free-bulk liquid state of **1** (sol. free) supported on quartz plate and in dichloromethane solution (soln) (c = 1 x 10^{-4} M, l = 1 mm, λ_{ex} = 375 nm) of **1** and **4**. (**b**) Comparison of the variation of emission spectra of **4** cast thin film by irradiating at 365 nm using a Xe lamp (100 W m^{-2}).

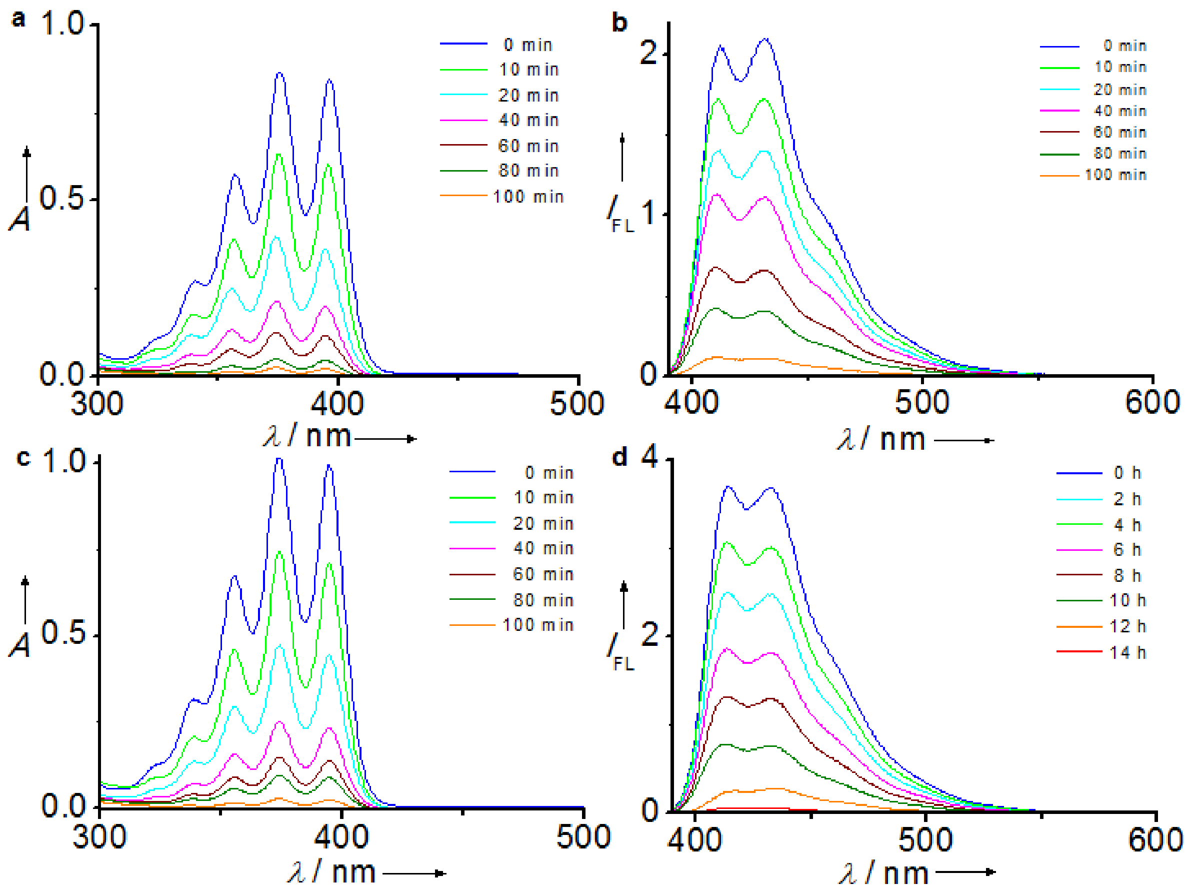

Supplementary Figure S6 | Fluorescence changes of 4 upon photo irradiation. Comparison of the variation of absorption (left; **a** and **c**) and emission (right; **b** and **d**) spectra of **4** in (**a**) and (**b**) dichloromethane and (**c**) and (**d**) in THF solutions by irradiating at 365 nm using a Xe lamp (100 W m^{-2}).

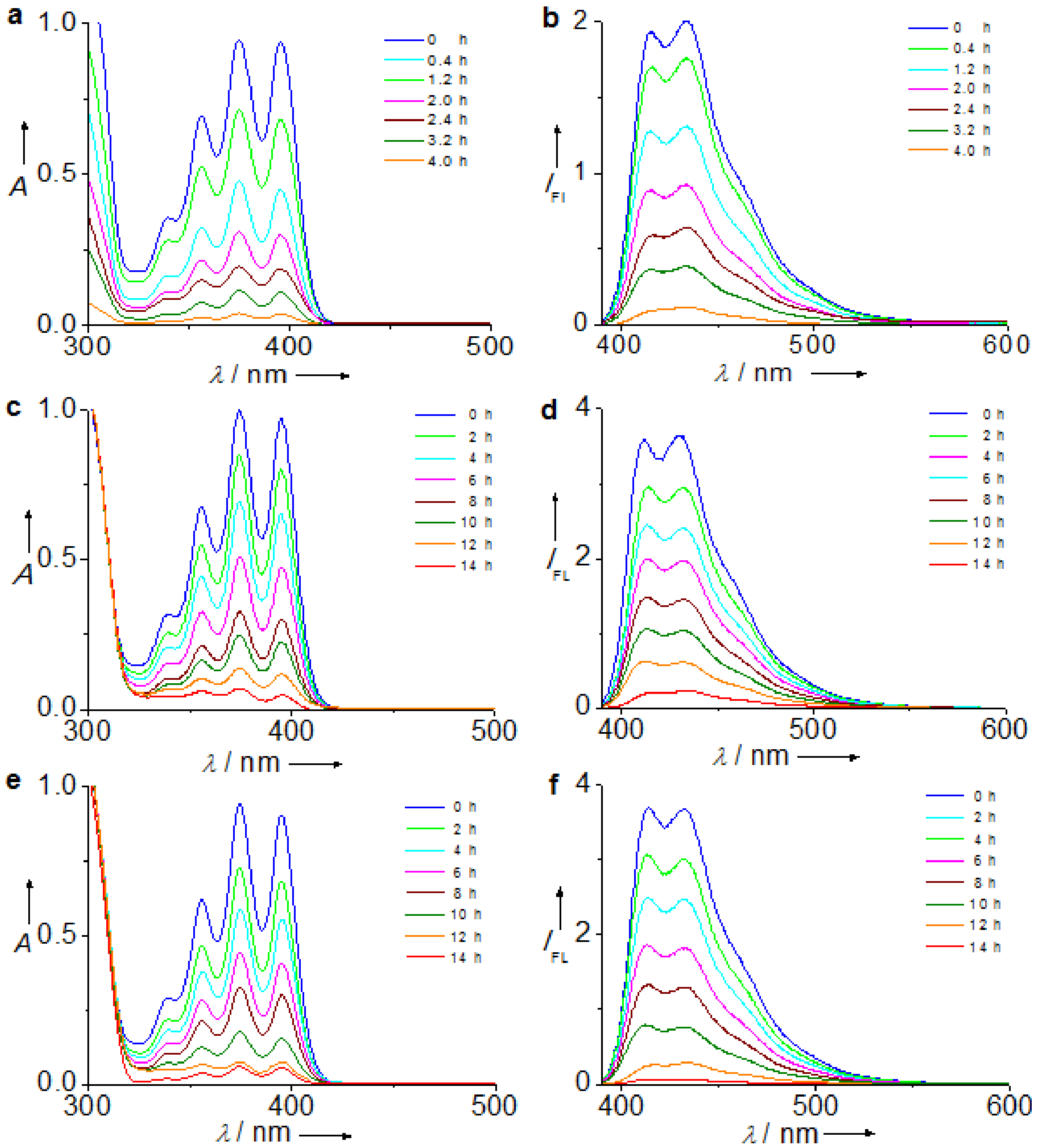

Supplementary Figure S7 | Fluorescence changes of 1 upon photo irradiation. Comparison of the variation of absorption (left; **a**, **c**, **e**) and emission (right; **b**, **d**, **f**) spectra of **1** in the (**a**) and (**b**) solvent-free films, (**c**) and (**d**) dichloromethane and (**e**) and (**f**) THF, with time upon irradiating at 365 nm using a Xe lamp (100 W m^{-2}).

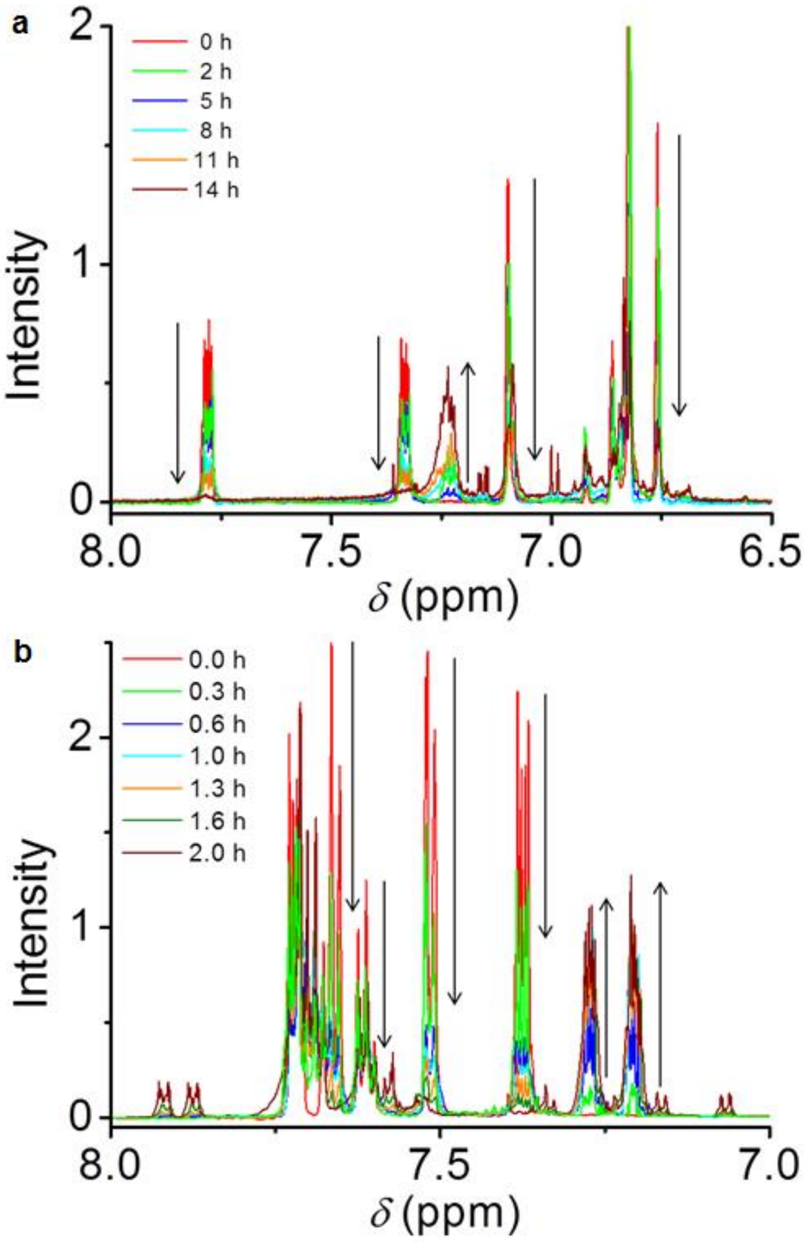

Supplementary Figure S8 | ^{1}H NMR changes of upon photo irradiation. Variation of the ^{1}H NMR signal intensity of aromatic protons of (**a**) **1** and (**b**) **4** in dichloromethane-d_2 solution from (**a**) 0 h to 14 h and (**b**) 0 h to 2 h irradiations, respectively, at 365 nm using a Xe lamp (100 W m^{-2}) at solvent-free film samples.

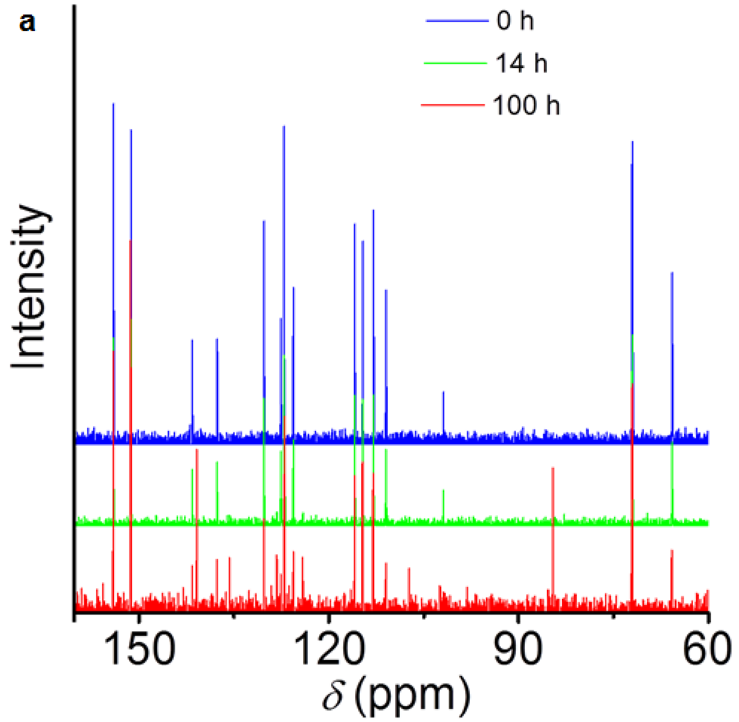

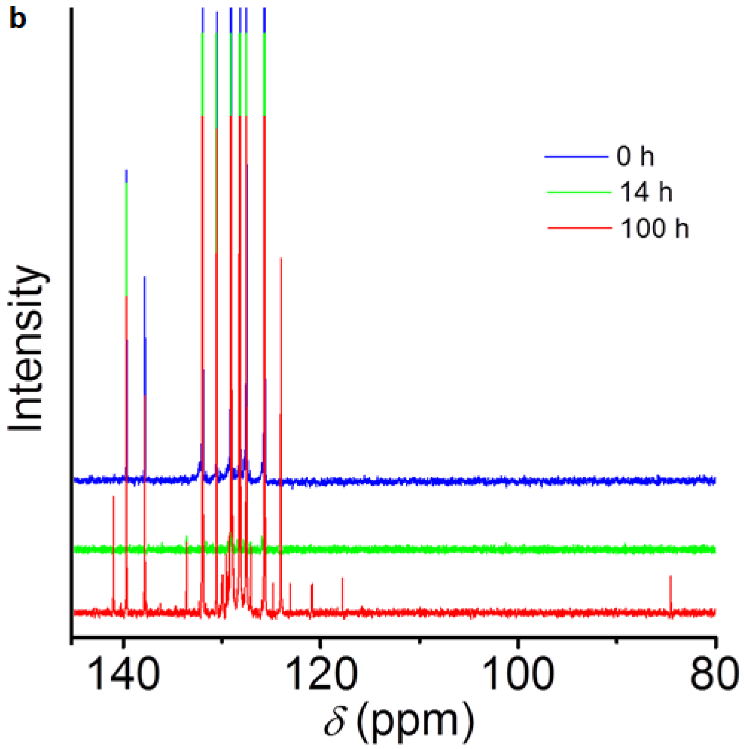

Supplementary Figure S9 | ^{13}C NMR changes upon photo irradiation. Variation of the ^{13}C NMR signal intensity (60-160 ppm) of (**a**) **1** (60-160 ppm) and (**b**) **4** (80-145 ppm) in dichloromethane-d_2 solution from 0 h to 100 h irradiations at 365 nm using a Xe lamp (100 W m^{-2}). The solutions used for ^{13}C experiments were more highly concentrated (20 mg/1g of CD_2Cl_2), hence longer photo irradiation time was required.

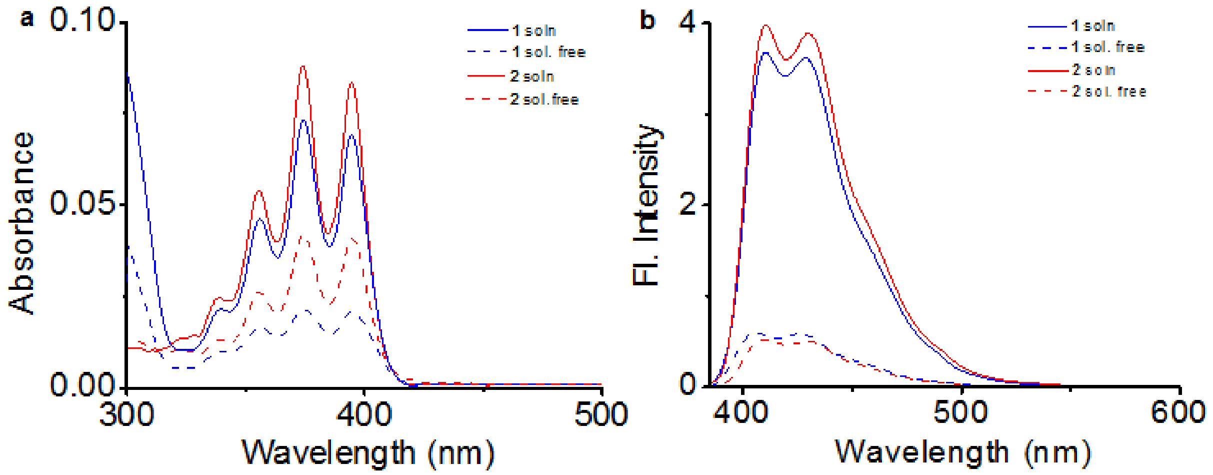

Supplementary Figure S10 | Absorption and emission spectra with real intensity. Raw data of (**a**) absorption spectra and (**b**) emission spectra of **1** (blue) and **2** (red) in solvent free film and in dichloromethane solution (c = 5 x 10^{-5} M, l = 1 mm, λ_{ex} = 375 nm).

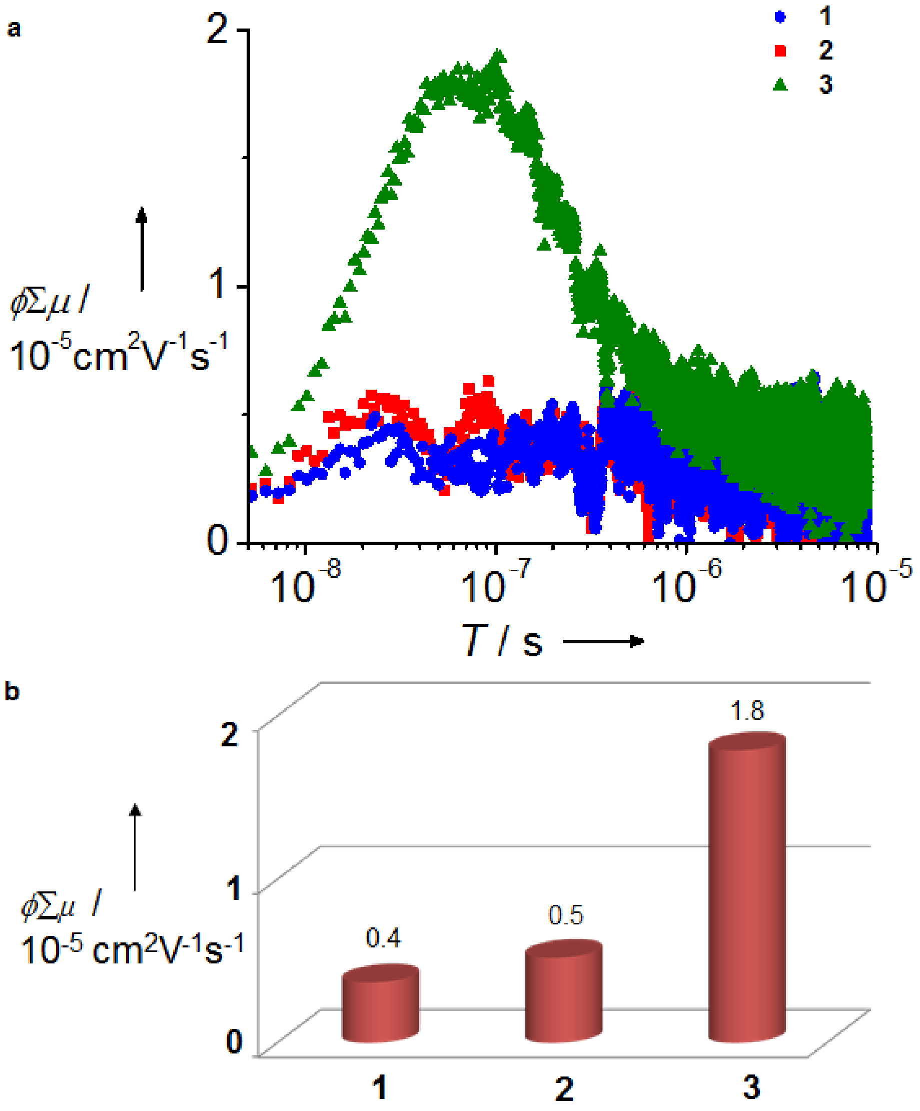

Supplementary Figure S11│Time resolved microwave conductivity studies. (**a**) FP-TRMC transient decay profiles of liquid **1** (●), **2** (■) and solid **3** (▲) (x-axis is in logarithmic time scale) and (**b**) comparison of the transient maxima of **1**, **2** and **3** under an excitation at λ = 355 nm by 1.8 x 10^{16} photons cm^{-2} pulse^{-1}. Transient photoconductivity ($\phi\Sigma\mu$, the product of the quantum efficiency of the charge carrier generation (ϕ) and the sum of the nanometer-scale charge-carrier mobilities ($\Sigma\mu$)), has been measured by irradiating with a λ = 355 nm laser pulse. The transient photoconductivity was low for both **1** and **2** (in the order of 10^{-6} cm^2 V^{-1} s^{-1}), clearly indicating that core isolation effectively controls the optoelectronic properties. The $\phi\Sigma\mu$ values exhibited by **1** and **2** are 3-4 times lower than for the reference molecule **3** which assembles in solid state at room temperature.

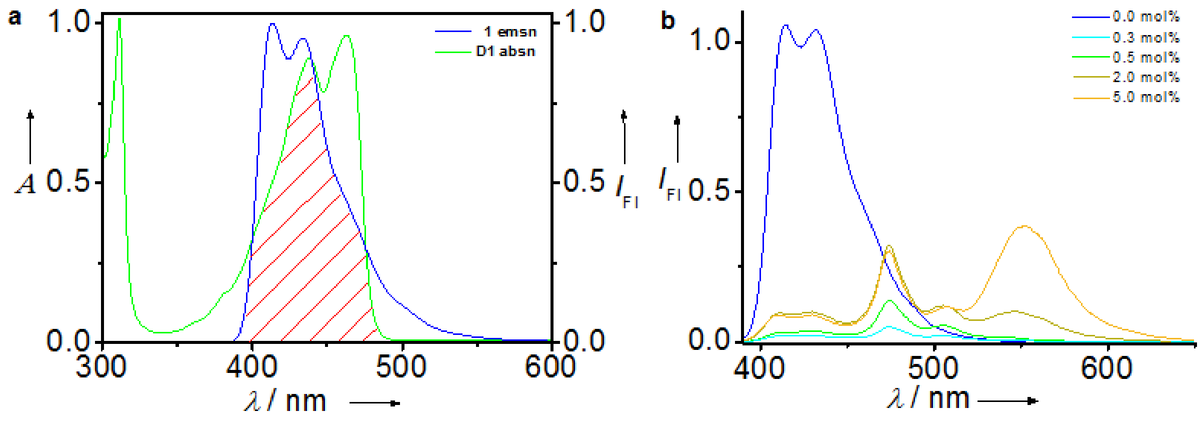

Supplementary Figure S12 | Energy transfer studies of 1 and D1. (**a**) Spectral overlap between the emission of **1** (blue) and the absorption of **D1** (green) in dichloromethane ((c = 5 x 10^{-5} M, l = 1 mm, λ_{ex} = 375 nm for **1**, 460 nm for **D1**)). (**b**) Raw data of the emission spectral changes of **1** upon increasing the concentration of **D1** (0.3-5.0 mol%) (λ_{ex} = 350 nm) in the solvent-free state supported on a quartz plate.

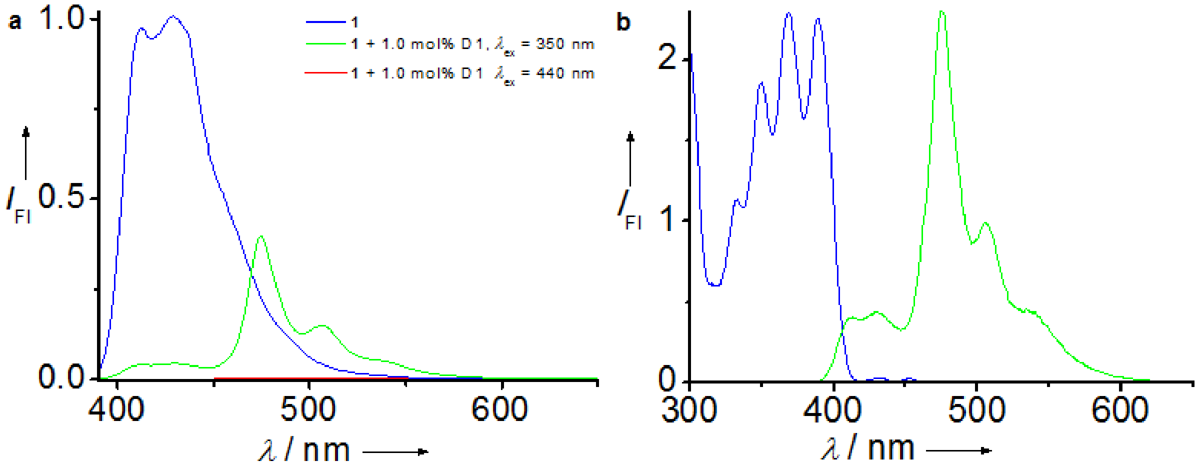

Supplementary Figure S13│Energy transfer studies of 1 and 1.0 mol% of D1. (**a**) Fluorescence spectra (blue) of **1** (λ_{ex} = 350 nm), in the presence of 1.0 mol% of **D1** upon exciting at λ = 350 nm (green) and λ = 440 nm (red) in the solvent-free state supported on quartz plate. (**b**) Normalised excitation (blue) (λ_{em} = 475 nm) and fluorescence (green) spectra of **1** in the presence of 1.0 mol% of **D1** upon exciting at λ = 350 nm in the solvent-free state supported on quartz plate.

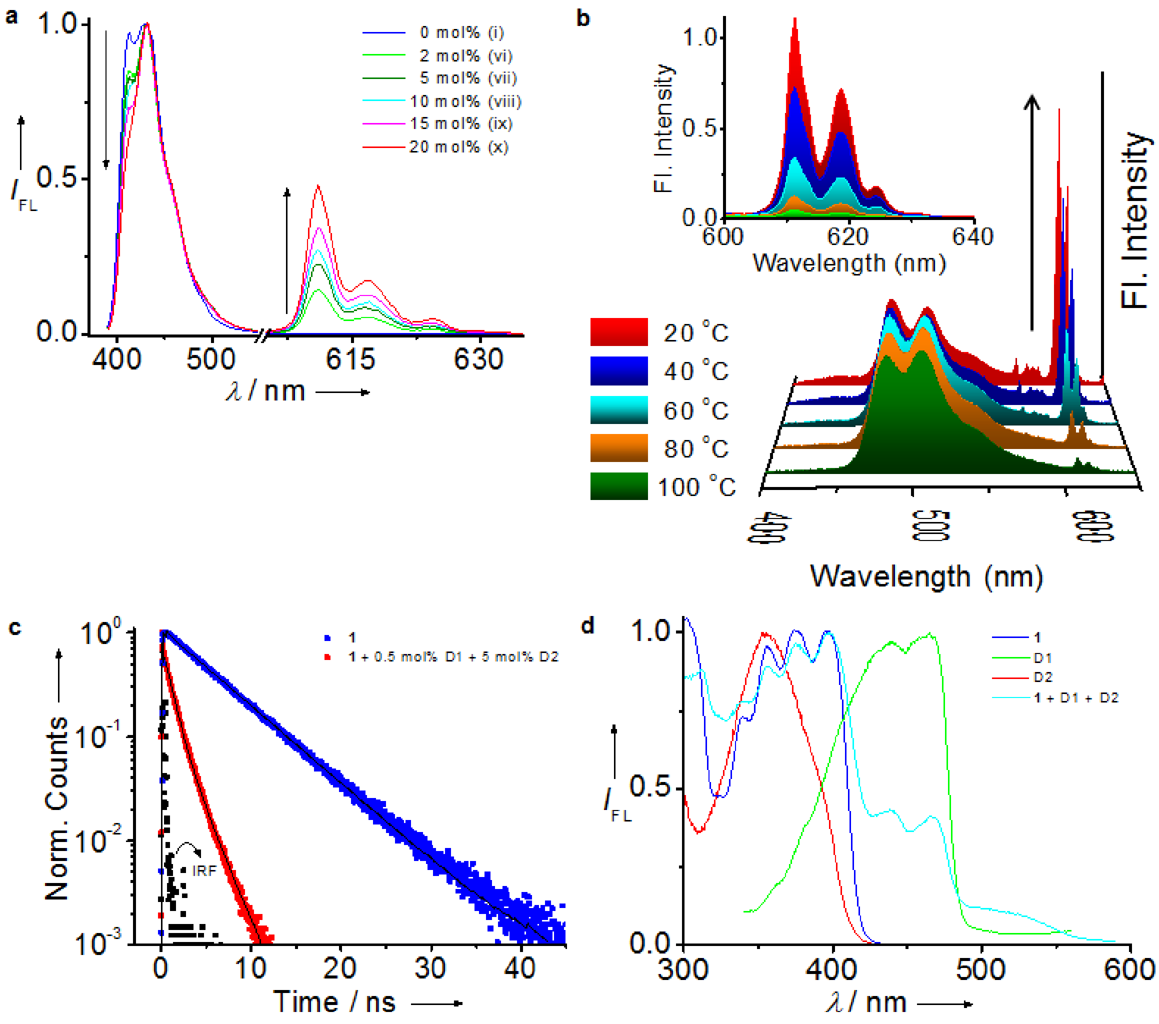

Supplementary Figure S14 | Thermoresponsive doping studies. (**a**) Normalized emission spectra of **1** in the presence of varying mol% of **D2** (λ_{ex} = 375 nm) in the solvent-free state supported on quartz plate. (**b**) Thermoreversible luminescence spectral changes of the composite of **1**, **D1** and **D2** upon cooling, supported on a quartz plate (λ_{ex} = 375 nm); inset shows the corresponding emission changes from 600-640 nm. (**c**) The fluorescence lifetime decay profiles of **1** in the absence (blue) and presence (red) of dopants **D1** (0.5 mol%), **D2** (5 mol%), monitored at 430 nm (λ_{ex} = 377 nm) in the solvent-free state supported on quartz plate. (IRF = Instrument Response Function). (**d**) Normalized excitation spectra of **1** (—), **D1** (—), **D2** (—) and a combination of **1**, **D1** (0.5 mol %) and **D2** (5.0 mol %) (—) supported on quartz plate (λ_{em} = 430 nm for **1**, 475 nm for **D1**, 610 nm for **D2** and 610 nm for composite).

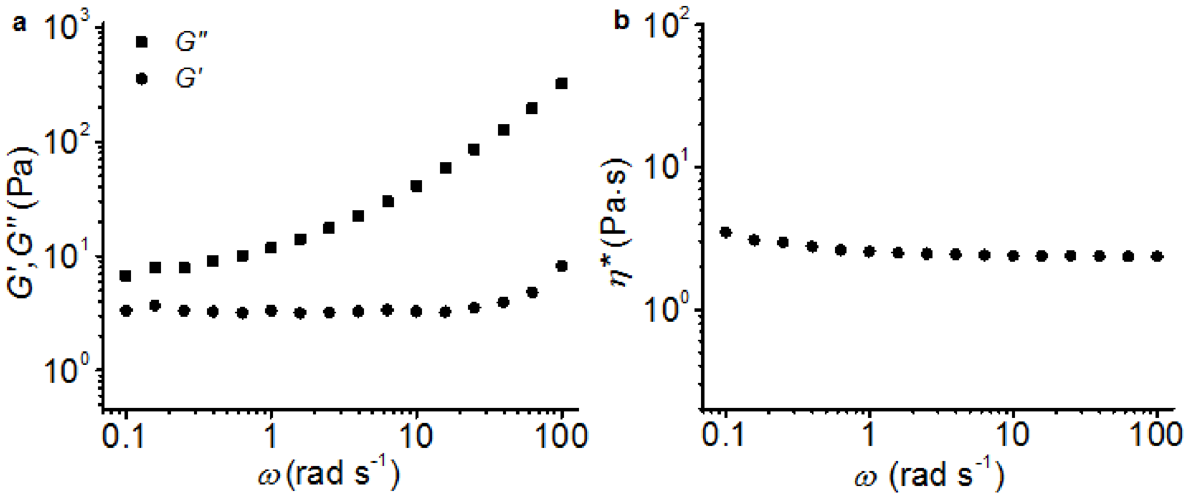

Supplementary Figure S15 | Rheology of composites. Variation of (**a**) storage modulus (*G'*) (●) and loss modulus (G″) (■) versus angular frequency on double logarithmic scale, and (**b**) complex viscosity (*η**) versus angular frequency on double logarithmic scale for composite of **1**, **D1** (0.5 mol%) and **D2** (5 mol%), shear strain (*γ*) = 0.1.

Supplementary Table S1. Fluid and optical characteristics of anthracenes **1** and **2**.

**Compound**	**Glass Transition Temperature** *T*g (°C)	**Molar Heat Capacity** C_{mol} (J mol^{-1} K^{-1})	**Complex Viscosity** (ω = 10 rad s^{-1}), η* (Pa·s)	**Refractive Index** nD^{20}	**Absolute QY** (Φ_f) sol.-free bulk at rt	**Absolute QY** (Φ_f) sol.-free bulk at 77K
**1**	-59.6	1955.1	0.28	1.516	0.54	0.65
**2**	-32.4	389.6	84.0	1.532	0.55	0.64

Supplementary Table S2. Photophysical parameters of anthracenes **1-3** in dilute CH_2Cl_2 solution.

**Compound**	$\lambda_{\mathbf{max}}$ (abs)	ε (M^{-1} cm^{-1})	**log** ε	$\lambda_{\mathbf{max}}$ (em)	**Absolute QY** (Φ_f) CH_2Cl_2 (1 x 10^{-5} M)
**1**	374	15,300	4.18	413	0.97
**2**	374	16,000	4.20	410	0.96
**3**	375	15,400	4.28	413	0.98

Supplementary Table S3. CIE coordinate values of liquid anthracenes **1 (i)**, **2** and doped hybrid systems **ii-xv**.

Trial	CIE coordinate values	Trial	CIE coordinate values
**1 (i)**	(0.11, 0.24)	**viii**	(0.46, 0.17)
**2**	(0.11, 0.23)	**ix**	(0.55, 0.21)
**ii**	(0.20, 0.32)	**x**	(0.61, 0.27)
**iii**	(0.26, 0.50)	**xi**	(0.51, 0.40)
**iv**	(0.30, 0.45)	**xii**	(0.46, 0.42)
**v**	(0.33, 0.51)	**xiii**	(0.40, 0.44)
**vi**	(0.29, 0.10)	**xiv**	(0.34, 0.45)
**vii**	(0.37, 0.14)	**xv**	(0.27, 0.51)

Supplementary Methods

Synthesis of alkyl and alkyloxybenzyl bromide.[13,17]

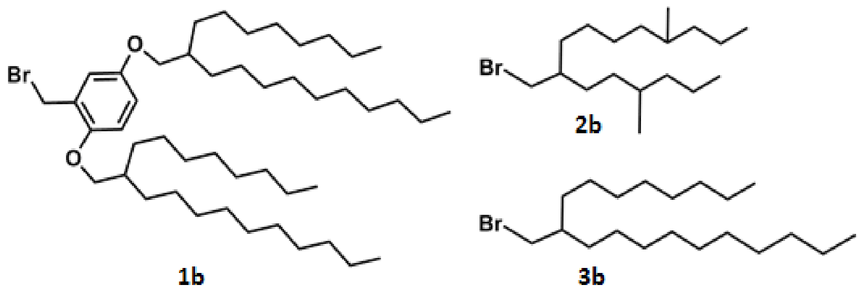

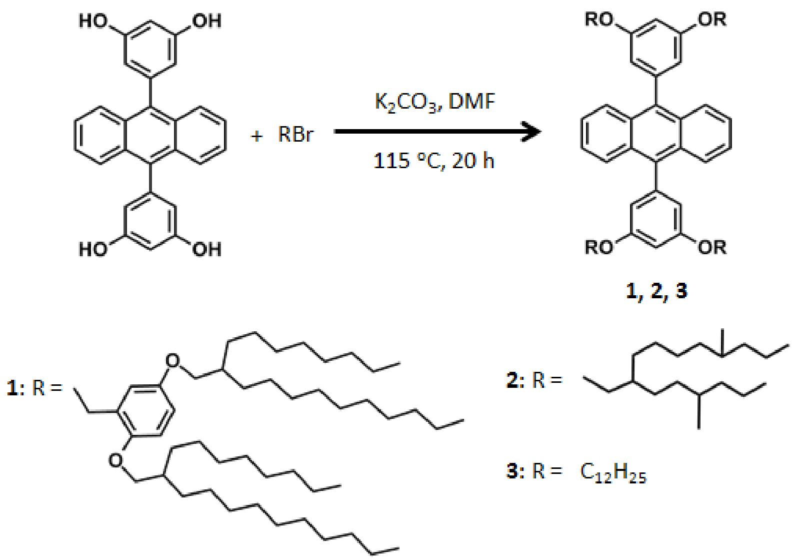

To a mixture of triphenylphosphine (PPh_3, 7.87 g, 30 mmol) and carbon tetrabromide (CBr_4, 5.97 g, 18 mmol) in 40 mL anhydrous THF, 15 mmol of the corresponding alkyl or alkyloxybenzyl alcohol dissolved in 10 mL anhydrous THF was added. The mixture was stirred for 30 min at room temperature under argon. The reaction mixture was filtered to remove insoluble solids, and THF was removed under reduced pressure. The residue was washed with *n*-hexane and filtered. Evaporation of *n*-hexane under reduced pressure followed by column chromatography (*n*-hexane) over silica gel yielded the pure compounds (**1b**-**3b**) as

colorless oily liquid in high yield (>90%). **1b:** ^{1}H NMR (400 MHz, $CDCl_3$): δ 3.70-3.16 (*m*, 2H), 2.10-0.55 (*m*, 35 H). **2b:** ^{1}H NMR (400 MHz, $CDCl_3$): δ 3.55-3.35 (*d*, 2H), 1.66-1.52 (1H), 1.46-1.15 (32H), 1.00-0.75 (*t*, 6H). **3b:** ^{1}H NMR (400 MHz, $CDCl_3$), δ 6.89-6.93 (*s*, 1H, Ar), 6.77-6.84 (*m*, 2H, Ar), 4.53-4.60 (*s*, 2H, Br-CH_2-Ar), 3.75-3.92 (*m*, 4H, OCH_2), 1.76-1.87 (*m*, 2H, C$H(CH_2)_3$), 1.10-1.47 (*m*, 64H, CH_2), 0.85-0.97 (*m*, 12H, CH_3); MALDI-TOF-MS, (matrix-dithranol): calculated for $C_{47}H_{87}BrO_2$ 764.10; found 763.95 [M^+].
